# Cross‐cultural factorial validation of the Clinical Interview Schedule – Revised (CIS‐R); findings from a nationally representative survey (EMPIRIC)

**DOI:** 10.1002/mpr.1428

**Published:** 2014-01-30

**Authors:** Jayati Das‐Munshi, Erico Castro‐Costa, Michael E. Dewey, James Nazroo, Martin Prince

**Affiliations:** ^1^ Section of Epidemiology, PO 60, Department of Health Service and Population Research Institute of Psychiatry, King's College London De Crespigny Park London SE5 8AF UK; ^2^ Centro de Pesquisa de Rene Rachou, Fiocruz Belo Horizonte MG Brazil; ^3^ Department of Sociology, School of Social Sciences University of Manchester Manchester M60 1QD UK

**Keywords:** EMPIRIC, CIS‐R, common mental disorders, ethnic minorities, confirmatory factor analysis

## Abstract

The Clinical Interview Schedule – Revised (CIS‐R) has been widely adopted across cultures to assess common mental disorders. We assessed the factorial validity of the CIS‐R across ethnic minority groups, using data from a nationally representative survey conducted in England in 2000. The sample comprised White British (*n* = 837), Irish (*n* = 733), Black Caribbean (*n* = 694), Bangladeshi (*n* = 650), Indian (*n* = 643) and Pakistani (*n* = 724) respondents. Ordered logistic regression determined the reporting of CIS‐R symptoms. Principal components analysis (PCA) determined the underlying construct of the CIS‐R in White British participants. These factor solutions were then assessed for “best fit” using confirmatory factor analyses (CFAs) across all ethnic groups.

In ordered logistic regression analyses, there was heterogeneity in the reporting of worries, phobias, panic and somatic symptoms across ethnic minority groups relative to the White British group. “Best” fit solutions confirmed through CFA were models where all symptoms were allowed to vary across ethnic groups, or models where an underlying “depression‐anxiety” construct was held invariant while “somatic symptoms” were permitted to vary across groups, although differences between models assessed were slight.

In conclusion, there may be benefits in assessing the functioning of certain CIS‐R items within specific cultural contexts to ensure adequate face validity of the CIS‐R. Copyright © 2014 John Wiley & Sons, Ltd.

## Background

Culturally informed presentations of mental distress continue to provide challenges to psychiatric diagnoses (Dimsdale *et al*., 2007). Epidemiological studies have suggested that the common mental disorders are universal phenomena both in international and transcultural settings (Simon *et al*., 1999; Simon *et al*., 2002; Weich *et al*., 2004), however, it is widely held that the expression and explanations accorded to these presentations may differ across cultures, and may not be fully captured through western diagnostic practises (Kleinman, 1977), which could account for reported differences in prevalence rates of common mental disorders between ethnic minority groups (Nazroo, 1999; Demyttenaere *et al*., 2004; Weich *et al*., 2004; Breslau *et al*., 2005; Asnaani *et al*., 2010).

The term “idioms of distress” refers to the mode by which psychological distress is experienced and expressed, and is associated with “culturally pervasive values, norms, generative themes, and health concerns” (Nichter, 1981). An epidemiological understanding of the phenomenonology of the common mental disorders across cultures is crucial, as differing idioms of distress may impact on patterns of help‐seeking, as well as influence the recognition of psychological morbidity by health care providers (Simon *et al*., 1999).

The Clinical Interview Schedule – Revised (CIS‐R) is a structured validated instrument which has been used to assess the prevalence of common mental disorders (Lewis *et al*., 1992). Although it has been used in a number of contexts to examine the prevalence of common mental disorders across ethnic minority groups (Sproston and Nazroo, 2002) or even to act as a “gold standard” against which other instruments are assessed (Patel *et al*., 2008), there have been very few studies which have directly assessed its psychometric properties within a cross‐cultural context. One previous study examined the factor structure of the CIS‐R using samples derived from primary care across four different international settings, however smaller sample sizes resulted in low prevalence symptoms being discarded from this analysis (Jacob *et al*., 1998).

With this in mind, we used data from a large, representative community‐based national survey containing a “boosted” sample (over‐sampled) of ethnic minority people living in England, the Ethnic Minorities Psychiatric Illness Rates in the Community Survey (EMPIRIC) (Sproston and Nazroo, 2002), to assess the cross‐cultural factorial validity of the CIS‐R. The CIS‐R was originally developed and validated in primary care samples from London and Santiago (Lewis *et al*., 1992), although has subsequently been adapted for use in many other cultural contexts (Sproston and Nazroo, 2002; Wickramasinghe *et al*., 2002; Patel *et al*., 2008; Jacob *et al*., 2010). An assumption therefore is that the underlying factor structure or construct validity of the common mental disorders as assessed by the CIS‐R is similar across cultures. We wished to assess this assumption using confirmatory factor analysis (CFA) approaches. The advantage of the current analysis was in the use of data from a nationally representative community‐based sample of people representing five of the main ethnic minority groups living in England, as well as a White British group. The administration of the CIS‐R was similar across all ethnic groups, and wherever possible interviewers were matched by gender and ethnicity to that of study participants. In addition, as the sample was “boosted” or over‐sampled for each of the ethnic groups at the sampling stages, adequate numbers of people in each of the groups were interviewed to permit stability in factor analysis estimates.

The main objectives of this study are to assess: (1) if the reporting of the 14 symptoms on the CIS‐R varies across ethnic minority groups; (2) if the underlying factor structure of the common mental disorders as assessed by the CIS‐R is similar across ethnic groups; (3) if the “fit” of the CIS‐R across ethnic groups improves when “somatic symptoms” are allowed to vary whilst “depression‐anxiety” symptoms are held invariant, thus assessing the hypothesis that there is a universal underlying “depression”/”anxiety” construct to the common mental disorders, although somatic symptom expression may vary according to cultural context (Weich *et al*., 2004).

## Methods

### Study design and participants

The data for analysis derived from the EMPIRIC, which was a nationally representative survey of ethnic minority groups living in private households in Britain, in 2000 (Sproston and Nazroo, 2002). The EMPIRIC was a follow‐on survey of the previous 1998 and 1999 Health Survey for England (HSE) (Sproston and Nazroo, 2002). Individuals who had participated in the HSE and had consented to being re‐contacted at a later date, comprised the sample. Of the 92% of individuals who had consented to taking part in a follow‐up survey, complete interviews were achieved in 68%, resulting in a final sample size of 4281 participants (Sproston and Nazroo, 2002). Respondents comprised people from the five main ethnic minority groups living in Britain (Irish, Indian, Pakistani, Bangladeshi, Black Caribbean), as well as a White British group, aged 16 to 74.

Sampling was based on a probabilistic selection of postcode sectors as the primary sampling unit, determined by the proportion of ethnic minority people residing within each sector (Erens and Primatesta, 2001; Sproston and Nazroo, 2002). Sample weightings took account of the unequal probability of selection for each postcode sector as well as the probability of household selection within each sector. Up to four people per household could be interviewed, therefore weighting also took account of the varying probability of selection within each household, using the Kish grid method (Kish, 1965). Survey weights were determined using logistic regression analysis for predictors of non‐response (Sproston and Nazroo, 2002).

Ethnicity for White British, Indian, Bangladeshi, Pakistani and Black Caribbean people was determined using a self‐report question which approximated closely to the criteria previously used in the 1991 UK Census. As the Irish ethnicity category did not exist in the 1991 Census criteria. ethnicity of this group was determined by the country of birth of the respondent and his/her parents (Sproston and Nazroo, 2002).

A professional translation agency translated survey materials into Urdu, Hindi, Gujarati, Bengali and Punjabi. The material was checked for linguistic equivalence by a researcher fluent in the language, and then checked by experienced bilingual interviewers. Where survey participants could not speak English, interviews were undertaken by a trained lay interviewer fluent in the language of the respondent. Interviewers could read and speak Urdu, Hindi, Gujarati, Bengali or Punjabi, alongside English. As non‐English script cannot be incorporated in Computer Assisted Interviewing Procedures (CAPI) translated versions of the interviews were administered using a paper document along with a computer (Sproston and Nazroo, 2002).

### Measures

The CIS‐R (Lewis et al., 1992) [11] was used to assess common mental disorders. In the CIS‐R, 14 different symptom groups are enquired after in the previous month, focusing on symptoms experienced within the last week. The 14 symptoms enquired after were: (1) Somatic symptoms; (2) Fatigue; (3) Sleep problems; (4) Irritability; (5) Physical health worries; (6) Depression; (7) Depressive ideas; (8) Worry; (9) Anxiety; (10) Phobias; (11) Panic; (12) Compulsive behaviours; (13) Obsessive thoughts; (14) Forgetfulness/concentration problems. Scores on each symptom group ranged from 0 to 4 (and 0 to 5 for depressive ideas), with higher scores indicating higher levels of symptomatology.

Age (in years) and gender were included in analyses as confounding variables. In order to adjust for the possibility that groups more likely to suffer from common mental disorders would be also more likely to report symptoms, we also adjusted for overall CIS‐R scores in analyses. CIS‐R scores were entered into models as a continuous sum of total symptoms reported.

### Statistical analysis

#### Analysis plan

We first assessed the prevalence of symptom reporting to determine if rates of expressing somatic or anxiety/depressive symptoms in ethnic minority groups differed from that of the White British group. Then, using principal components analysis (PCA) we assessed the underlying factor structure of common mental disorders according to the CIS‐R in the White British population. To assess if the “fit” of the underlying factor structure as determined in the White British group was similar to that of the ethnic minority groups surveyed, we used CFA. CFA is a powerful technique which permits the examination or “fit” of known or established underlying factor structures in new populations, unlike PCA it is hypothesis‐driven rather than exploratory.

Finally, we assessed if the “fit” of the CIS‐R across ethnic groups improved when “somatic symptoms” were allowed to vary whilst “depression‐anxiety” symptoms were held invariant, using CFA.

#### Statistical methods

Regression analyses were performed in Stata 11 (2009). Weighting to correct for non‐response bias in the EMPIRIC compared to the 1998 and 1999 HSE was derived, and applied in all regression analyses (Erens and Primatesta, 1999, 2001; Sproston and Nazroo, 2002). These non‐response weights were derived using data from the prior HSE surveys, where data was available for responders and non‐responders (Sproston and Nazroo, 2002). Stepwise logistic regression techniques were used to determine significant demographic variables which were predictive of non‐response; these included a number of person‐level demographic variables, household‐level variables, and finally, National Health Service (NHS) region, which was the primary sampling unit. Non‐response weights also took into account interactions with ethnicity (Sproston and Nazroo, 2002).

Multivariable ordered logistic regressions were performed whereby scores on each of the 14 symptom groups on the CIS‐R were entered into models as ordinal dependent variables. Ethnicity, with the White British group as “reference” category, was the independent variable. Age, gender and total CIS‐R scores were entered into models as confounders.

PCA was based on a covariance matrix of tetrachoric correlations to reduce bias in the estimation of factor loading (Olsson, 1979). Oblique rotation was performed, with an eigenvalue of greater than one as the initial extraction criteria. Symptoms reported on the 14 subscales of the CIS‐R, were entered into models as ordered categorical variables, and the factor structure assessed on the White British group first.

To assess if the underlying latent traits as derived through PCA in the White British group varied in the same way across ethnic minority groups, CFA was used. CFA can be used to assess whether a scale measures the same trait dimension, in the same way, when applied in qualitatively distinct groups (Reise *et al*., 1993). We wished to test for measurement invariance (Sorbom, 1974); that is, if the best factor solution was related to the latent trait or traits in the same way across the White British and five ethnic minority groups. In this way, factor solutions as determined through PCA on the White British sample were assessed for goodness‐of‐fit, using CFA, across each of the ethnic groups surveyed.

The values of *X* ^2^ was used to initially test absolute model fit, with a smaller *X* ^2^ value indicating a better fit. However the assumptions of *X* ^2^ break down with large datasets which are not normally distributed (Byrne, 2001). Therefore goodness‐of‐fit indices were also used to estimate relative and absolute fit of models (Bollen and Long, 1993; Byrne, 2001). Goodness‐of‐fit indices used in this analysis included the Tucker–Lewis Index (TLI) (Tucker and Lewis, 1973), Akaike's Information Criterion (AIC) (Akaike, 1987), and the root mean square error of approximation (RMSEA) (Browne, 1990). TLI is not dependent on sample size, and assesses proposed models against a null hypothesis model of independence. TLI values range from 0.00 to 1.00, with values > 0.90 suggesting satisfactory fit (Tucker and Lewis, 1973), AIC assesses the measure of fit of models and also takes into account estimated parameters, penalizing for model complexity. Models with a lower AIC value suggest a better fit of the model to the data (Akaike, 1987). The RMSEA is sensitive to the number of parameters in the model and assesses how poorly the model fits the data. It has been suggested that RMSEA values < 0.05 indicate a “close” fit, from 0.05 to 0.08 indicate a “fair” fit, and between 0.05 to 0.08 suggest a “mediocre” fit (MacCallum *et al*., 1996).

To assess models with constraints, analyses were performed in AMOS 18 ([Link]) on unweighted data, as it was not possible to perform these in STATA 11. Sensitivity analyses comparing models without constraints on unweighted data in AMOS gave estimates within three decimal places of estimates derived using weighted data, in STATA.

## Results

### Demographic features of sample

In total, the sample comprised 837 White British, 733 Irish, 694 Black Caribbean, 650 Bangladeshi, 643 Indian and 724 Pakistani respondents. Of 3444 study participants from one of the five ethnic minority groups, 41% were second generation. Interviews were conducted in English for 85% of the sample; the following languages were used for the remainder: 0.9% Gujarati, 0.3% Hindi, 6.2% Punjabi, 1.5%, Urdu, 8.1% Bengali, 2% Sylheti. Of the sample 54% was female, and 33% of the sample were in social class 4 and 5, with the ethnic minority groups tending to be of a lower social class than the White British group. Full discussion of the demographic features of the sample is available in the main report (Sproston and Nazroo, 2002).

### Symptom reporting on the CIS‐R across ethnic minority groups

Table 1 displays the results of ordinal logistic regression analysis of reporting any of the 14 symptoms on the CIS‐R, taking the White British group as the reference. Relative to the White British reference group, Bangladeshi, Indian and Pakistani people were each more likely to be in a higher category with respect to reporting somatic symptoms or symptoms relating to physical health worries. Conversely, these three groups were less likely to endorse symptoms relating to phobias and worries relative to the White British group. Compared to the White British reference population the Black Caribbean group were 1.67 times more likely to report physical health worries [95% confidence interval (CI): 1.17, 2.39], for each unit increase in the reporting of this symptom group. All of the groups except the Irish group were less likely than the White British group to report symptoms relating to irritability. Symptom reporting was fairly similar in the Irish group relative to the White British group.

**Table 1:** Ordered logistic regression of symptoms by ethnic group; White British group is the reference

		Ethnic minority group:				
		Irish	Black Caribbean	Bangladeshi	Indian	Pakistani
	Symptom prevalence^a^	OR (95% CI)	OR (95% CI)	OR (95% CI)	OR (95% CI)	OR (95% CI)
Depressive symptoms	24%	0.87 (0.65, 1.17)	1.00 (0.75, 1.34)	1.15 (0.83, 1.58)	1.16 (0.87, 1.56)	1.14 (0.86, 1.51)
Depressive ideas	17%	0.99 (0.68, 1.44)	1.06 (0.72, 1.55)	0.99 (0.65, 1.50)	1.03 (0.70, 1.53)	1.08 (0.73, 1.58)
Somatic symptoms	13%	1.02 (0.66, 1.57)	0.97 (0.65, 1.45)	1.86**(1.22, 2.83)	1.77** (1.19, 2.63)	1.82**(1.23, 2.71)
Fatigue symptoms	38%	0.88 (0.69, 1.12)	0.91 (0.72, 1.16)	0.62** (0.46, 0.83)	0.87 (0.67, 1.14)	0.83 (0.64, 1.08)
Forgetfulness/concentration problems	21%	1.32 (0.97, 1.79)	1.34 (0.99, 1.81)	1.40 (0.98, 1.98)	1.05 (0.76, 1.44)	0.95 (0.68, 1.33)
Sleep problems	36%	1.16 (0.91, 1.47)	0.83 (0.65, 1.06)	0.97 (0.74, 1,27)	0.77* (0.59, 1.00)	0.93 (0.72, 1.19)
Irritability	36%	1.19 (0.95, 1.48)	0.64*** (0.51, 0.81)	0.28*** (0.21, 0.39)	0.60*** (0.47, 0.77)	0.39***(0.30, 0.50)
Physical health worries	17%	0.97(0.66, 1.42)	1.67** (1.17, 2.39)	2.56*** (1.76, 3.73)	1.75** (1.20, 2.57)	2.18***(1.53, 3.09)
Worries	31%	1.01 (0.79, 1.30)	0.88 (0.69, 1.12)	0.41***(0.30, 0,57)	0.68** (0.52, 0.89)	0.47***(0.35, 0.62)
Anxieties	16%	1.34 (0.95, 1.87)	0.90 (0.63, 1.28)	0.75 (0.49, 1.16)	0.86 (0.59, 1.25)	0.84 (0.59, 1.21)
Phobias	10%	1.37 (0.94, 1.99)	1.24 (0.86, 1.81)	0.30***(0.17, 0.54)	0.62* (0.40, 0.97)	0.47***(0.31, 0.73)
Panic	4%	1.63 (0.81, 3.30)	1.04 (0.49, 2.23)	2.17*(1.03, 4.59)	1.25 (0.60, 2.59)	0.93 (0.45, 1.93)
Compulsions	8%	1.03 (0.64, 1.67)	1.36 (0.88, 2.10)	0.70 (0.39, 1.25)	1.30 (0.80, 2.10)	1.26 (0.80, 2.01)
Obsessions	9%	0.93 (0.60, 1.44)	1.36 (0.89, 2.08)	0.65 (0.38, 1.12)	1.06 (0.66, 1.71)	1.13 (0.73, 1.74)

Note: Models have adjusted for gender age and total symptom count and take into account survey weights for non‐response. OR, odds ratio; CI, confidence interval.

* *p* < 0.05.

** *p* < 0.01.

*** *p* < 0.001 (Wald tests).

a Weighted proportion of respondents in full sample (*n* = 4281) endorsing one or more symptoms.

### Principal components analysis (PCA)

Table 2 displays the results of PCA performed on the White British group in the EMPIRIC. Using eigenvalues > 1 as extraction criteria, PCA suggested a three factor solution, which comprised a “depression‐anxiety” factor (Factor 1: forgetfulness/concentration problems, sleep problems, irritability, depression, worry, anxiety, obsessive thoughts and depressive ideas), a “somatic symptoms factor” (Factor 2: somatic symptoms, fatigue, physical health worries, panic) and a third factor which comprised phobias and compulsive behaviours. The scree plot (available from the authors) was also suggestive of a two‐factor and a one‐factor solution. The two‐factor solution comprised an underlying “Depression‐Anxiety” construct (Factor 1) which consisted of: forgetfulness/concentration problems, sleep problems, irritability, depression, worry, phobias, compulsive behaviours, obsessive thoughts and depressive ideas and a second underlying “Somatic symptoms” construct, which comprised: somatic symptoms, fatigue, physical health worries, anxiety and panic (Table 2). A one‐factor solution was also thought to potentially account for the data (Table 2). For the two‐factor and three‐factor solutions the first factor (representing an underlying “depression‐anxiety” construct) and the second factor (representing a “somatic symptoms” construct) were moderately correlated. For the three‐factor solution the third factor, which consisted of phobias and compulsive behaviours, had low correlations with both the “depression‐anxiety” construct and the “somatic symptoms” construct.

**Table 2:** One‐, two‐ and three‐factor solutions from exploratory factor analysis of the CIS‐R in the White British sample (*N* = 837)

	One‐factor solution	Two‐factor solution		Three‐factor solution		
		Factor 1	Factor 2	Factor 1	Factor 2	Factor 3
		Depression‐anxiety	Somatic	Depression‐anxiety	Somatic	Phobic compulsions
Somatic symptoms	0.72	0.05	**0.75**	0.24	**0.61**	0.05
Fatigue	0.79	0.15	**0.72**	0.42	**0.55**	−0.03
Forgetfulness/concentration problems	0.76	**0.63**	0.21	**0.60**	0.07	0.21
Sleep problems	0.57	**0.41**	0.22	**0.54**	0.10	−0.01
Irritability	0.68	**0.44**	0.31	**0.63**	0.15	−0.04
Physical health worries	0.59	−0.34	**1.02**	−0.13	**0.91**	0.05
Depression	0.79	**0.64**	0.23	**0.84**	0.05	−0.05
Worry	0.80	**0.45**	0.44	**0.63**	0.27	0.01
Anxiety	0.79	0.33	**0.55**	**0.62**	0.36	−0.10
Phobias	0.56	**0.36**	0.26	−0.14	0.26	**0.79**
Panic	0.79	0.27	**0.61**	0.14	**0.51**	0.42
Compulsive behaviours	0.57	**0.65**	−0.03	0.10	−0.02	**0.79**
Obsessive thoughts	0.59	**1.00**	−0.35	**0.76**	−0.43	0.33
Depressive ideas	0.87	**0.67**	0.29	**0.87**	0.09	−0.02
Eigenvalues		7.11	1.15	7.11	1.15	1.03
Variance (%)		5.98	5.85	6.27	4.47	3.40
Correlation between factors						
1 and 2		0.59		0.54		
1 and 3				0.08		
2 and 3				−0.14		

Note: Bold typeface items indicate highest loadings across factors.

### Confirmatory factor analysis (CFA)

CFA of each of the three‐factor, two‐factor and one‐factor models were individually assessed; each will be considered in this section.

Table 3 displays results from the CFA for the three‐factor model; Model 1 displays loadings for the three‐factor solution with no constraints, Model 2 shows factor loadings where the underlying construct for somatic symptoms (Factor 2) has been allowed to vary across all ethnic groups while Factor 1 (anxiety‐depressive symptoms) and Factor 3 (phobic compulsions) have been constrained. Model 3 displays loadings for the three‐factor solution which was fully constrained. Comparative goodness‐of‐fit statistics have also been displayed in Table 3, for each model. As expected, the model with no constraints (i.e. all symptoms allowed to vary freely across ethnic groups) had the lowest AIC value and lowest chi‐squared value. The AIC deteriorated marginally (by 44.83) in Model 2 – where the depression‐anxiety and phobic‐compulsions constructs were fixed but the “somatic symptoms” construct was permitted to vary (Table 3). Compared to the fully constrained model, the AIC for the partially constrained model was closer to that of the model with no constraints (Table 3). Chi‐squared also showed a deterioration when comparing partially and fully constrained models to the unconstrained model, with a corresponding increase in degrees of freedom. The RMSEA was 0.03 across all models suggesting excellent fit. TLI suggested only a moderate fit, but of note remained broadly similar across models.

**Table 3:** Confirmatory factor analysis (CFA) of the CIS‐R in full sample; three‐factor solutions

	Factor 1: “Depression‐anxiety”								Factor 2: “Somatic symptoms”				Factor 3: “Phobic compulsions”	
	Forgetfulness/concentration problems	Sleep problems	Irritability	Depression	Worry	Anxiety	Obsessive thoughts	Depressive ideas	Somatic symptoms	Fatigue	Physical health worries	Panic	Phobias	Compulsive behaviours
*Model 1: No constraints; all symptoms permitted to vary*														
White British	1.00	1.09	1.28	1.22	1.54	1.07	0.62	1.25	1.00	2.29	0.60	0.40	1.00	1.01
Irish	1.00	1.09	1.06	0.92	1.32	1.03	0.58	0.96	1.00	2.62	0.64	0.60	1.00	0.96
Black Caribbean	1.00	1.20	1.05	0.99	1.30	0.78	0.54	0.96	1.00	2.59	0.92	0.38	1.00	1.53
Bangladeshi	1.00	1.03	0.73	1.00	1.11	0.64	0.48	0.96	1.00	1.68	1.02	0.47	1.00	1.49
Indian	1.00	1.09	0.91	1.06	1.26	0.85	0.68	1.09	1.00	1.98	0.80	0.39	1.00	1.28
Pakistani	1.00	1.24	1.02	1.06	1.11	0.84	0.76	1.07	1.00	1.75	0.86	0.33	1.00	1.50
*Goodness‐of‐fit statistics for Model 1*														
*χ* ^2^	2583.178													
	*p* < 0.001													
df	444													
AIC	2955.18													
TLI	0.85													
RMSEA	0.03													
***Model 2: Factor 1 and Factor 3 are constrained while Factor 2 is permitted to vary***														
White									1.00	2.32	0.59	0.40		
Irish									1.00	2.60	0.64	0.59		
Black Caribbean	1.00	1.14	1.00	1.05	1.27	0.86	0.61	1.06	1.00	2.58	0.91	0.38	1.00	1.31
Bangladeshi									1.00	1.68	1.02	0.46		
Indian									1.00	1.98	0.79	0.38		
Pakistani									1.00	1.75	0.86	0.33		
*Goodness‐of‐fit statistics for Model 2*														
*χ* ^2^	2708.01													
	*p* < 0.001													
df	484													
AIC	3000.01													
TLI	0.86													
RMSEA	0.03													
***Model 3: Fully constrained model; all symptoms have been constrained across the full sample***														
Overall sample	1.00	1.14	0.99	1.04	1.26	0.85	0.60	1.04	1.00	2.11	0.63	0.44	1.00	1.31
*Goodness‐of‐fit statistics for Model 3*														
*χ* ^2^	3025.66													
	*p* < 0.001													
df	499													
AIC	3287.66													
TLI	0.84													
RMSEA	0.03													

Table 4 displays the CFA loadings for the two‐factor solution. Model 1 displays the two‐factor solution with no constraints, in general the factor loadings for the “somatic symptoms” construct in the unconstrained model appeared to be quite variable across ethnic groups. Model 2 in Table 4 displays factor loadings for a partial invariance model where the depression‐anxiety construct has been constrained but somatic symptoms have been allowed to vary. Model 3 in Table 4 shows factor loadings for the fully constrained two‐factor solution. As with the three‐factor solution TLI and RMSEA did not vary a great deal between models, with RMSEA indicating an excellent fit across models and TLI a more moderate fit. AIC in the model where anxiety‐depression symptoms were constrained but somatic symptoms permitted to vary, deteriorated by 46.04 when compared to the unconstrained model, but was improved compared to the fully constrained model (difference of 203.23). Chi‐squared also showed a marginal deterioration comparing the fully constrained model to the partially constrained model and the partially constrained model to the unconstrained model (Table 4).

**Table 4:** Confirmatory factor analysis (CFA) of the CIS‐R in the full sample – two‐factor solutions

Factor 1: “Depression‐anxiety”										Factor 2: “Somatic symptoms”				
	Forgetfulness/concentration problems	Sleep problems	Irritability	Depression	Worries	Phobias	Compulsive behaviours	Obsessive thoughts	Depressive Ideas	Somatic symptoms	Fatigue	Physical health worries	Anxiety	Panic
*Model 1: No constraints; all symptoms permitted to vary*														
White British	1.00	1.08	1.27	1.21	1.53	0.35	0.39	0.62	1.24	1.00	2.31	0.60	1.05	0.39
Irish	1.00	1.08	1.05	0.91	1.31	0.48	0.46	0.58	0.95	1.00	2.60	0.62	1.02	0.58
Black Caribbean	1.00	1.20	1.05	0.98	1.29	0.39	0.59	0.54	0.96	1.00	2.58	0.91	0.78	0.38
Bangladeshi	1.00	1.01	0.73	0.99	1.10	0.28	0.40	0.48	0.95	1.00	1.67	1.01	0.63	0.45
Indian	1.00	1.10	0.91	1.07	1.27	0.29	0.39	0.68	1.09	1.00	1.97	0.81	0.86	0.40
Pakistani	1.00	1.25	1.03	1.08	1.12	0.36	0.52	0.77	1.08	1.00	1.74	0.87	0.85	0.34
*Goodness‐of‐fit statistics for model 1*														
*χ* ^2^	2690.84													
	*p* < 0.001													
df	456													
AIC	3038.84													
TLI	0.84													
RMSEA	0.03													
***Model 2: Partial invariance model; Factor 1 is constrained whilst Factor 2 is permitted to vary***														
White British										1.00	2.33	0.60	0.93	0.40
Irish										1.00	2.58	0.62	1.04	0.56
Black Caribbean	1.00	1.13	0.99	1.04	1.27	0.35	0.46	0.61	1.05	1.00	2.60	0.92	0.78	0.38
Bangladeshi										1.00	1.67	1.01	0.72	0.45
Indian										1.00	1.97	0.80	0.86	0.40
Pakistani										1.00	1.74	0.87	0.84	0.34
*Goodness‐of‐fit statistics for Model 2*														
*χ* ^2^	2816.88													
	*p* < 0.001													
df	496													
AIC	3084.88													
TLI	0.86													
RMSEA	0.03													
***Model 3: Fully constrained model; all symptoms have been constrained across the full sample***														
Overall sample	1.00	1.13	0.99	1.04	1.26	0.35	0.46	0.61	1.05	1.00	2.05	0.77	0.99	0.45
*Goodness‐of‐fit statistics for Model 3*														
*χ* ^2^	3060.11													
	*p* < 0.001													
df	516													
AIC	3288.11													
TLI	0.84													
RMSEA	0.03													

Table 5 displays factor loadings for the one‐factor solution in the full sample, without any constraints. Factor loadings across ethnic groups were fairly similar for the one factor solution. As expected, chi‐squared and degrees of freedom deteriorated from one‐factor to two‐ and three‐factor solutions, although differences were not marked. AIC appeared to marginally improve from one‐factor > two‐factor > three‐factor solutions, whereas RMSEA and the TLI did not vary much between models (unconstrained solutions in Tables 3, 4, 5).

**Table 5:** Confirmatory factor analysis (CFA) of one‐factor solution in full sample; no constraints

	Worries	Anxieties	Phobias	Panic	Obsessive thoughts	Compulsive behaviours	Somatic symptoms	Fatigue	Forgetfulness/concentration problems	Sleep problems	Irritability	Physical health worries	Depression	Depressive ideas
White British	1.00	0.69	0.24	0.23	0.39	0.25	0.53	1.28	0.66	0.73	0.83	0.31	0.78	0.80
Irish	1.00	0.78	0.37	0.31	0.44	0.35	0.51	1.36	0.77	0.83	0.80	0.33	0.69	0.72
Black Caribbean	1.00	0.61	0.30	0.24	0.41	0.46	0.58	1.51	0.78	0.94	0.81	0.52	0.76	0.74
Bangladeshi	1.00	0.57	0.26	0.43	0.44	0.38	0.84	1.42	0.93	0.95	0.66	0.86	0.92	0.87
Indian	1.00	0.68	0.22	0.31	0.53	0.31	0.66	1.33	0.79	0.87	0.71	0.53	0.84	0.86
Pakistani	1.00	0.77	0.32	0.35	0.68	0.45	0.86	1.55	0.91	1.13	0.91	0.75	0.97	0.96
*Goodness‐of‐fit statistics: One‐factor solution, no constraints*														
*χ* ^2^	2856.31													
	*p*<0.001													
df	462													
AIC	3192.31													
TLI	0.84													
RMSEA	0.03													

Partial measurement invariance models (two‐factor versus three‐factor solutions), where “somatic symptoms” were allowed to vary whilst the “depression‐anxiety” construct was held constant, were compared using goodness‐of‐fit statistics (Model 2; Tables 3 and 4). Again, whereas RMSEA, TLI and to an extent chi‐squared values were fairly similar across the two models, the three‐factor solution in which the “somatic symptoms” construct was permitted to vary had a marginally superior AIC value compared to the two‐factor solution where the “somatic symptoms” construct was allowed to vary. The AIC is likely to be more sensitive to small differences in studies with larger sample sizes so is a tougher test than the TLI or RMSEA.

## Discussion

### Principal findings

We used a combination of approaches to assess symptom reporting on the CIS‐R and the underlying factorial validity of the CIS‐R across the main ethnic minority groups living in England, alongside a White British group. Findings from the regression models suggested heterogeneity in the reporting of specific symptoms by ethnic minorities living in England, relative to the White British group. The only group which were an exception to this were people of Irish ethnicity, who in general share a similar language to the White British group and so due to semantic equivalence might be expected to be similar in the reporting of symptoms. Whereas some symptoms such as phobias, worries, irritability and fatigue were reported less frequently by some of the ethnic minority groups in the survey relative to the White British group, other symptoms such as “somatic symptoms” and “physical health worries” were reported more frequently, especially by Indian, Pakistani and Bangladeshi people within the sample. Whereas the regression analyses established how the reporting of symptoms on the CIS‐R across ethnic minority groups differed from a White British reference, the factor analyses established if the underlying factor structure for the common mental disorders – and the factorial validity of the CIS‐R – were similar across the ethnic groups surveyed. In addition, the CFA established if the “fit” of derived constructs could be improved across groups by taking into account the possibility that the underlying construct for “somatic symptoms” might vary across ethnic minority groups.

Exploratory factor analysis of the CIS‐R in the White British group suggested a one‐factor solution as well as a two‐factor solution (comprising an underlying “depression‐anxiety” construct as well as a “somatic symptoms” construct) which were correlated. This underlies the observation that the “internalizing disorders” may reflect a common underlying factor which consists of both anxiety‐depressive symptoms as well as somatic symptoms (Stein *et al*., 2010). Of note, a three‐factor solution was also identified which consisted of compulsive behaviours and phobias. This third factor was poorly correlated with both the “depression‐anxiety” factor and the “somatic symptoms” factor. The latter finding is in keeping with previous analyses which have suggested that two underlying factors may account for the internalizing disorders, namely a “fear” factor and an “anxious‐misery” factor (Krueger, 1999), or that a third “phobic avoidance” factor may exist, which is poorly correlated with an anxiety‐depression factor (cited in Goldberg, 2010).

When assessed in the full ethnic minority sample using CFA, all three (unconstrained) models were, broadly similar in their fit statistics, with the three‐factor solution showing a marginally improved fit over the two‐factor and one‐factor solutions. To assess if the factor structure of the CIS‐R was the same across all ethnic groups, we compared fit statistics for fully constrained models to models without constraints. Our findings suggested that when loadings were set to be equal across samples for each of the one‐factor, two‐factor or three‐factor solutions, the fit deteriorated, compared to models where loadings were allowed to vary. This would support the assertion that underlying constructs assessed by the CIS‐R are not identical across the ethnic groups surveyed. However, we also assessed the hypothesis that allowing an underlying “somatic symptom” construct to vary across ethnic groups, whilst holding a “depression‐anxiety” construct as invariant would improve the “fit” of the CIS‐R. We found that the fit of models where “somatic symptoms” were allowed to vary was slightly improved over fully constrained models, suggesting that variability in the reporting of the underlying “somatic symptoms” construct across ethnic groups (whilst holding the “depression‐anxiety” construct as invariant) proved a marginally improved fit of the CIS‐R over fully constrained models.

### Strengths and weaknesses

Major strengths of this study included the use of a large community based sample in the analysis, which used a stratified sampling methodology that oversampled for ethnic minority group, as well as the use of a standardized administered diagnostic tool to elicit common mental disorder dimensional symptom counts across ethnic groups surveyed (Sproston and Nazroo, 2002). Interviewers were matched to study participants by gender and language and this would have improved response rates and increased the validity of instruments used in the survey (Sproston and Nazroo, 2002). Previous epidemiological research has suggested that observed differences in somatization may be a part reflection of underlying differences in service provision (Simon *et al*., 1999; Escobar and Gureje, 2007), where somatization may reflect a “ticket of admission” amongst people presenting to primary care (Goldberg and Bridges, 1988). Our study would have avoided this potential selection bias by using a community‐based sample of individuals living in private households. The large sample size would have ensured that derived estimates from factor analysis would have been reasonably stable. In addition, our study used current measures of reported symptomatology rather than life‐time reports, which have been shown in previous epidemiological research to be associated with recall bias and inconsistent reporting of somatic symptoms (Simon *et al*., 1999; Escobar and Gureje, 2007). A further advantage was that we were able to look at the full range of common mental disorder psychopathology and not just limit our analyses to depression and/or “threshold” conditions. Previous studies have been arguably limited by such approaches, as in the arena of transcultural psychiatry many expressions of psychological morbidity may not be captured by traditional Western diagnostic constructs (Kleinman, 1987).

A number of limitations affected our study. There is a longstanding debate within the cross‐cultural literature around *etic* and *emic* approaches to understanding expressions of distress across cultures (Kleinman, 1987; Littlewood, 1990). Whereas *etic* approaches favour quantitative methods and implicitly assume universalistic explanations of mental disorder, *emic* approaches tend to focus on local meanings that favour a relativistic understanding of psychological distress, with meaning and explanations grounded in local cultural beliefs and practices. *Etic* approaches allow comparisons between cultures, although risk committing “category fallacy” errors, whereby Western diagnostic constructs are reified and applied to other settings where they do not have relevance (Kleinman, 1977, 1987).

Whilst this study sought to avoid “category fallacy” errors by analysing dimensional symptom “counts”, rather than using categorical diagnoses, the analysis was still conducted within an *etic* framework. Although this was a relative strength in allowing a comparison of the groups surveyed, the main drawback is that certain types of expressions of distress would not have been captured by the 14 subsections of the CIS‐R. Previously, the qualitative phase of the EMPIRIC attempted to understand the metaphors or idioms used by survey participants to articulate psychological distress Nazroo and O'Connor, 2002). The findings from this qualitative study suggested that some types of symptoms are *not* universal across all ethnic minority groups, and so may have been missed by the itemized approach used in the CIS‐R (Nazroo and O'Connor, 2002).

### Relationship to previous research

These findings should be seen in light of previous research. Our results are somewhat consistent with another study which examined the factor structure of common mental disorders in Santiago, Harare, Rotherhithe and Ealing (Jacob *et al*., 1998), which confirmed a similar underlying factor structure for common mental disorders across international centres. This study found that a partially constrained one‐factor solution where symptoms relating to worry, anxiety and concentration problems were unconstrained (or permitted to vary), provided the best fit for the CIS‐R across centres (Jacob *et al*., 1998). As we had an adequate sample size we were able to use all 14 symptom groups on the CIS‐R for our analysis – the authors in the previous analyses had to discard “low prevalence” symptoms, which were phobias, panic, obsessions and compulsions (Jacob *et al*., 1998). Inclusion of these symptoms in our analyses led to the different findings in our study compared to this previous study.

A more recent analysis examined the CIS‐R across a number of international primary and community‐based datasets (Jacob *et al*., 2010). This analysis suggested that a two‐factor model (composed of a “depression” and an “anxiety” construct) provided a marginally better fit than a one‐factor solution, although when fully constrained, the analyses suggested that neither of these solutions fitted well across study sites (Jacob *et al*., 2010). The findings of this study also suggested that the underlying “anxiety” and “depression” constructs across sites were highly correlated (Jacob *et al*., 2010), supporting a one‐factor solution. We also found that underlying latent traits representing a mixed “depression‐anxiety” construct and a second “somatic symptoms” construct were moderately correlated, although allowing the “somatic symptoms” construct to vary improved the fit of the models across ethnic groups, compared to fully constrained models.

The underlying factor solutions presented in the current study differ from that of the “tripartite model” for common mental disorders, proposed by Clark and Watson (1991). In this conceptualization, a three‐factor solution comprising specific anxiety, specific depression, and a third “distress” factor underpin the common mental disorders Clark and Watson, 1991). This model has found support in a recent CFA of the CIS‐R, by Skapinakis *et al*. (2011), on a Greek sample of 16 to 18 year olds. In the analysis by Skapinakis *et al*. (2011) questions relating to somatic symptoms on the CIS‐R were excluded. This may be why the findings of the study by Skapinakis *et al*. (2011) differed from our study. In addition, the sample in the study by Skapinakis *et al*. (2011) differed from our sample by ethnicity and age, which may further account for differences in findings.

In addition, previous work conducted in international settings has indicated that whilst there are large cross‐national differences at which specific types of symptoms are endorsed at the same severity level of depression, the underlying latent structure of reported symptoms remains similar between different centres (Simon *et al*., 2002; Castro‐Costa *et al*., 2007). Our findings are consistent with the possibility that the prevalence of symptoms varies across ethnic groups and also with the observation that the level at which symptoms are endorsed may differ across ethnic groups, and may reflect real cultural or linguistic differences in the interpretation and understanding of individual items (Castro‐Costa *et al*., 2007), or be embedded in specific social contexts (Littlewood, 1990). The findings from the CFA lead us to suggest that there could be a universal (“depression‐anxiety”) construct that underpins the expression of psychological distress, but this is not inconsistent with the possibility that the social and cultural context in which people live play a significant part in modulating the expression and meanings attached to such distress (Littlewood, 1990), which may have been reflected in the variability of the reporting of somatic symptoms across groups, as found in this study. A future line of enquiry may be to assess the impact of language and generational status, as proxy measures for acculturation, on the reporting of symptoms. In addition, gender and social class could have an impact on the reporting of symptoms, and should be considered in future research.

The findings differ when compared to the World Health Organization (WHO) study of psychological problems in primary care, which found that whilst the prevalence of depression varied across 10 international sites, the prevalence of somatic presentations showed a strong correlation with the type of health provision service offered at each site (Simon *et al*., 1999). After controlling for potential confounding factors, the prevalence of three or more somatic symptoms at each centre did not vary significantly, with a strong and consistent relationship between depression and unexplained somatic symptoms across all centres (Simon *et al*., 1999). The authors concluded that somatic symptoms are a core feature of depressive syndromes, with variations in prevalence partially accounted for by “facultative somatisation” (Goldberg and Bridges, 1988), or health‐seeking behaviour differences (Simon *et al*., 1999). Unlike the WHO international study, we had the advantage of a randomly sampled community‐based survey, which should have eliminated any over‐reporting of symptoms as a function of “facultative somatization”. It is noteworthy that despite this design feature, we were able to detect clear differences in the prevalence of types of (somatic or otherwise) symptoms reported by each ethnic minority group, after accounting for overall level of morbidity.

In addition, the findings of the present analysis are in keeping with those from the qualitative phase of the EMPIRIC (Nazroo and O'Connor, 2002). The investigators of the qualitative phase concluded that the articulation of psychological distress appeared to be broadly universal across all groups surveyed although the “*fit*” of some symptoms was “*less good for some cultural groupings than others*” (Nazroo and O'Connor, 2002).

### Implications for future use of the CIS‐R in other cultures

The analyses presented here support the assertion that the CIS‐R retains factorial validity across ethnic minority groups, as constraining the depression‐anxiety factor whilst allowing “somatic symptoms” to vary across groups improved the fit of models compared to models which were fully constrained. This suggests that an underlying “depression‐anxiety” construct retains measurement invariance across ethnic groups, although there is variability in the reporting of somatic symptoms across cultures. In previous validation studies, investigators found that removing some of the symptoms from the CIS‐R improved the psychometric properties of the instrument. For example, in a study from Sri Lanka it was found that dropping items relating to phobias and sleep improved the cross‐cultural validity of the CIS‐R (Wickramasinghe *et al*., 2002). Our study suggests that although there are similarities in the underlying factor structure of the common mental disorders (and especially in a “depression‐anxiety” construct) across cultures, there may be benefits to assessing the functioning of some CIS‐R items within specific cultural contexts to ensure adequate face validity.

## Funding

Dr Jayati Das‐Munshi is sponsored by a Medical Research Council training fellowship. Dr Castro‐Costa is supported by the Programa Nacional de Pós‐doutorado em Saúde –PNDS. The funding bodies played no part in the study design, analysis of the data or in the preparation of the report.

## References

[mpr1428-bib-0001] Akaike H. (1987) Factor analysis and AIC. Psychometrika, 52(3), 317–332.

[mpr1428-bib-0002] Asnaani A. , Richey J.A. , Dimaite R. , Hinton D.E. , Hofmann S.G. (2010) A cross‐ethnic comparison of lifetime prevalence rates of anxiety disorders. Journal of Nervous Mental Disorders, 198(8), 551–555. 10.1097/NMD.0b013e3181ea169f PMC2931265 20699719

[mpr1428-bib-0003] Bollen K. , Long J. (1993) Testing Structural Equation Models, Newbury Park, CA, Sage.

[mpr1428-bib-0004] Breslau J. , Kendler K.S. , Su M. , Gaxiola‐Aguiler S. , Kessler R.C. (2005) Lifetime risk and persistence of psychiatric disorders across ethnic groups in the United States. Psychological Medicine, 35(3), 317–327. 15841868 10.1017/s0033291704003514 PMC2748985

[mpr1428-bib-0005] Browne M. (1990) MUTMUM PC: User's Guide, Columbus, OH, Ohio State University.

[mpr1428-bib-0006] Byrne B.M. (2001) Structural Equation Modelling with AMOS: Basic Concepts, Applications, and Programming, Englewood Cliffs, NJ, Lawrence Erlbaum Associates.

[mpr1428-bib-0007] Castro‐Costa E. , Dewey M. , Stewart R. , *et al* (2007) Prevalence of depressive symptoms and syndromes in later life in ten European countries: the SHARE study. British Journal of Psychiatry, 191(5), 393–401. 17978318 10.1192/bjp.bp.107.036772

[mpr1428-bib-0008] Clark L.A. , Watson D. (1991) Tripartite model of anxiety and depression: psychometric evidence and taxonomic implications. Special Issue on Diagnoses, Dimensions, and DSM‐IV: The Science of Classification. Journal of Abnormal Psychology, 100(3), 316–336. 1918611 10.1037//0021-843x.100.3.316

[mpr1428-bib-0009] Demyttenaere K.B.R. , Posada‐Villa J. , *et al* (2004) Prevalence, severity, and unmet need for treatment of mental disorders in the world health organization world mental health surveys. JAMA: The Journal of the American Medical Association, 291(21), 2581–2590. 15173149 10.1001/jama.291.21.2581

[mpr1428-bib-0010] Dimsdale J.E. , Patel V. , Xin Y. , Kleinman A. (2007) Somatic presentations – a challenge for DSM V. Psychosomatic Medicine, 69, 829.

[mpr1428-bib-0011] Erens B. , Primatesta P. , Health Survey for England (1999) Cardiovascular Disease '98, London, The Stationery Office.

[mpr1428-bib-0012] Erens B. , Primatesta P. , GP Health Survey for England 1999 (2001) The Health of Minority Ethnic Groups, London, The Stationery Office.

[mpr1428-bib-0013] Escobar J.I. , Gureje O. (2007) Influence of cultural and social factors on the epidemiology of idiopathic somatic complaints and syndromes. Psychosomatic Medicine, 69(9), 841–845. 18040091 10.1097/PSY.0b013e31815b007e

[mpr1428-bib-0014] Goldberg D. (2010) Psychometric aspects of anxiety and depression In Goldberg D. et al. (eds) Depression and Generalized Anxiety Disorder: Refining the Research Agenda for DSM‐V, pp. 109–123, Arlington, VA, American Psychiatric Association.

[mpr1428-bib-0015] Goldberg D.P. , Bridges K. (1988) Somatic presentations of psychiatric illness in primary care setting. Journal of Psychosomatic Research, 32(2), 137–144. 3042995 10.1016/0022-3999(88)90048-7

[mpr1428-bib-0016] IBM Corporation . (n.d.) IBM‐SPSS‐AMOS‐17, Route 100, Somers, NY, IBM Corporation.

[mpr1428-bib-0017] Jacob K.S. , Everitt B.S. , Patel V. , Weich S. , Araya R. , Lewis G.H. (1998) The comparison of latent variable models of non‐psychotic psychiatric morbidity in four culturally diverse populations. Psychological Medicine, 28, 142–152. 10.1017/s0033291797005710 9483690

[mpr1428-bib-0018] Jacob K.S. , Prince M. , Goldberg D. (2010) Confirmatory factor analysis of mental disorders across cultures In Goldberg D. *et al* (eds) Depression and Generalized Anxiety Disorder: Refining the Research Agenda for DSM‐V, pp. 191–210, Arlington, VA, American Psychiatric Association.

[mpr1428-bib-0019] Kish L. (1965) Survey Sampling, London, Wiley.

[mpr1428-bib-0020] Kleinman A. (1977) Depression, somatization and the new cross‐cultural psychiatry. Social Science and Medicine, 11, 3–10. 887955 10.1016/0037-7856(77)90138-x

[mpr1428-bib-0021] Kleinman A. (1987) The role of culture in cross‐cultural research on illness. British Journal of Psychiatry, 151, 447–454. 3447661 10.1192/bjp.151.4.447

[mpr1428-bib-0022] Krueger R.F. (1999) The structure of common mental disorders. Archives of General Psychiatry, 56(10), 921–926. 10530634 10.1001/archpsyc.56.10.921

[mpr1428-bib-0023] Lewis G. , Pelosi A.J. , Araya R. , Dunn G. (1992) Measuring psychiatric disorder in the community: a standardized assessment for use by lay interviewers. Psychological Medicine, 22, 465–486. 1615114 10.1017/s0033291700030415

[mpr1428-bib-0024] Littlewood R. (1990) From categories to contexts: a decade of the “New Cross‐Cultural Psychiatry”. British Journal of Psychiatry, 156, 308–327. 2189524 10.1192/bjp.156.3.308

[mpr1428-bib-0025] MacCallum R. , Browne M.W. , Sugawara H.M. (1996) Power analysis and determination of sample size for covariance structure modeling. Psychological Methods, 1(2), 130–149.

[mpr1428-bib-0026] Nazroo J. , O'Connor W. (2002) Idioms of mental distress In O'Connor W., Nazroo J. (eds) Ethnic Differences in the Context and Experience of Psychiatric Illness: A Qualitative Study, pp. 29–39, London, The Stationery Office.

[mpr1428-bib-0027] Nazroo J.Y. (1999) Ethnicity and Mental Health: Findings from a National Community Survey, London, Policy Studies Institute.

[mpr1428-bib-0028] Nichter M. (1981) Idioms of Distress: Alternatives in the Expression of Psychosocial Distress: A Case Study from South India. Culture, Medicine and Psychiatry, 5(4), 379–408. 10.1007/BF00054782 7326955

[mpr1428-bib-0029] Olsson U. (1979) Maximum likelihood estimation of the polychoric correlation coefficient. Psychometrika, 44(4), 443–460.

[mpr1428-bib-0030] Patel V. , Araya R. , Chowdhary N. , *et al* (2008) Detecting common mental disorders in primary care in India: a comparison of five screening questionnaires. Psychological Medicine, 38(2), 221–228. 18047768 10.1017/S0033291707002334 PMC4959557

[mpr1428-bib-0031] Reise S.P. , Widaman K.F. , Pugh R.H. (1993) Confirmatory factor analysis and item response theory: two approaches for exploring measurement invariance. Psychological Bulletin, 114(3), 552–566. 8272470 10.1037/0033-2909.114.3.552

[mpr1428-bib-0032] Simon G. , Goldberg D. , VonKorff M. , Ustun T.B. (2002) Understanding cross‐national differences in depression prevalence. Psychological Medicine, 32(4), 585–594. 12102373 10.1017/s0033291702005457

[mpr1428-bib-0033] Simon G.E. , VonKorff M. , Piccinelli M. , Fullerton C. , Ormel J. (1999) An international study of the relation between somatic symptoms and depression. New England Journal of Medicine, 341, 1329–1335. 10536124 10.1056/NEJM199910283411801

[mpr1428-bib-0034] Skapinakis P. , Anagnostopoulos F. , Bellos S. , Magklara K. , Lewis G. , Mavreas V. (2011) An empirical investigation of the structure of anxiety and depressive symptoms in late adolescence: cross‐sectional study using the Greek version of the revised Clinical Interview Schedule. Psychiatry Research, 186(2–3), 419–426. 20846727 10.1016/j.psychres.2010.08.023

[mpr1428-bib-0035] Sorbom D. (1974) A general method for studying differences in factor means and factor structure between groups. British Journal of Mathematical and Statistical Psychology, 27, 229–239.

[mpr1428-bib-0036] Sproston J. , Nazroo J. (2002) Ethnic Minority Psychiatric Illness Rates in the Community (EMPIRIC): Quantitative Report, London, The Stationery Office.

[mpr1428-bib-0037] Stata Corp . (2009) Stata Statistical Software: Release 11, College Station, TX, StataCorp LP.

[mpr1428-bib-0038] Stein D.J. , Patel V. , Heinze G. (2010) Commentary on “Confirmatory Factor Analysis of common mental disorders across cultures” In Goldberg D. *et al* (eds) Diagnostic Issues in Depression and Generalized Anxiety Disorder: Refining the Research Agenda for DSM‐V, pp. 211–216, Arlington, VA, American Psychiatric Association.

[mpr1428-bib-0039] Tucker L. , Lewis C. (1973) A reliability coefficient for maximum likelihood factor analysis. Psychometrika, 38, 1–10.

[mpr1428-bib-0040] Weich S. , Nazroo J. , Sproston K. , *et al* (2004) Common mental disorders and ethnicity in England: the EMPIRIC study. Psychological Medicine, 34, 1543–1551. 15724884 10.1017/s0033291704002715

[mpr1428-bib-0041] Wickramasinghe S.C. , Rajapakse L. , Abeysinghe R. , Prince M. (2002) The Clinical Interview Schedule – Sinhala version: validation in a community setting in Sri Lanka. International Journal of Methods in Psychiatric Research, 11(4), 169–177. 12459820 10.1002/mpr.134 PMC6878291

